# Circular RNA‐based neoantigen vaccine for hepatocellular carcinoma immunotherapy

**DOI:** 10.1002/mco2.667

**Published:** 2024-07-29

**Authors:** Fei Wang, Guang Cai, Yingchao Wang, Qiuyu Zhuang, Zhixiong Cai, Yingying Li, Shaodong Gao, Fang Li, Cuilin Zhang, Bixing Zhao, Xiaolong Liu

**Affiliations:** ^1^ The United Innovation of Mengchao Hepatobiliary Technology Key Laboratory of Fujian Province Mengchao Hepatobiliary Hospital of Fujian Medical University Fuzhou P. R. China; ^2^ Fujian Provincial Clinical Research Center for Hepatobiliary and Pancreatic Tumors Fuzhou P. R. China; ^3^ Mengchao Med‐X Center Fuzhou University Fuzhou P. R. China; ^4^ School of Basic Medical Sciences Fujian Medical University Fuzhou P. R. China

**Keywords:** circRNA, hepatocellular carcinoma, immunotherapy, neoantigen, vaccine

## Abstract

mRNA vaccines are regarded as a highly promising avenue for next‐generation cancer therapy. Nevertheless, the intricacy of production, inherent instability, and low expression persistence of linear mRNA significantly restrict their extensive utilization. Circular RNAs (circRNAs) offer a novel solution to these limitations due to their efficient protein expression ability, which can be rapidly generated in vitro without the need for extra modifications. Here, we present a novel neoantigen vaccine based on circRNA that induces a potent anti‐tumor immune response by expressing hepatocellular carcinoma‐specific tumor neoantigens. By cyclizing linearRNA molecules, we were able to enhance the stability of RNA vaccines and form highly stable circRNA molecules with the capacity for sustained protein expression. We confirmed that neoantigen‐encoded circRNA can promote dendritic cell (DC) activation and enhance DC‐induced T‐cell activation in vitro, thereby enhancing T‐cell killing of tumor cells. Encapsulating neoantigen‐encoded circRNA within lipid nanoparticles for in vivo expression has enabled the creation of a novel circRNA vaccine platform. This platform demonstrates superior tumor treatment and prevention in various murine tumor models, eliciting a robust T‐cell immune response. Our circRNA neoantigen vaccine offers new options and application prospects for neoantigen immunotherapy in solid tumors.

## Graphical abstract

This study developed a novel circRNA‐based neoantigen vaccine. In vitro experiments showed that circRNA encoding neoantigens can promote DC activation and enhance DC‐induced T‐cell activation in vitro, thereby enhancing T‐cell killing of tumor cells. The circRNA neoantigen vaccine exhibited potent therapeutic and prophylactic effects in diverse murine tumor models by eliciting a robust T‐cell‐mediated immune response.

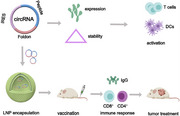

## 1 INTRODUCTION

Immunotherapy has become an effective anti‐tumor strategy for a variety of malignant tumors, including immune checkpoint inhibition and chimeric antigen receptor T‐cell therapy, but there are still shortcomings, such as low response rate, single target, high side effects, and easy relapse. More effective immunotherapies are being sought to treat patients with a variety of cancers. Among the various cancer immunotherapies in clinical trials, cancer vaccines are one of the most promising.^1^, ^2^ Neoantigens are novel antigens that are produced by tumor cells due to a range of tumor‐specific changes, including genomic mutations, aberrant RNA splicing, abnormal post‐translational modifications, and the incorporation of viral open reading frames (ORFs).^3^ Neoantigen‐based tumor therapeutic vaccines have achieved surprising clinical efficacy in a variety of tumors and have shown good application prospects.^4^

As a new therapeutic modality, personalized neoantigen tumor vaccine is still immature and faces many obstacles and challenges. Neoantigen‐based tumor vaccines are categorized into several types, predominantly including peptide/protein vaccines, dendritic cell (DC) vaccines, and nucleic acid vaccines, which encompass both DNA and mRNA vaccines.^5^ Among these subtypes of cancer vaccines, RNA‐based vaccines stand out as particularly promising. They are capable of rapidly expressing antigens within the cytoplasm, leading to robust immune activation. Additionally, they offer the advantage of avoiding the risks associated with genome integration and T‐cell tolerance.^6^, ^7^ mRNA neoantigen vaccine is a versatile and powerful platform that has been widely shown to be a promising immunotherapy strategy.^7^ Recently, BioNTech's personalized mRNA vaccine, autogene cevumeran (BNT122), has demonstrated potential in phase I clinical trials in pancreatic cancer.^8^

However, mRNA produced by in vitro transcription (IVT) has a relatively short half‐life in cells, so additional nucleotide modification is required to improve its stability, and current modification strategies, including nucleoside modifications, methylguanosine cap incorporation and codon optimization, significantly increase the cost of RNA vaccine production, but there was only a modest improvement in RNA stability. Therefore, alternative approaches that enhance the stability of RNA molecules and increase the duration of its encoded protein expression could aid in unlocking the full potential of RNA vaccines in cancer therapy.

Circular RNAs (circRNAs) are a significant group of non‐coding RNAs that form covalently closed loops. These molecules are generated in eukaryotic cells through a unique RNA splicing process known as back splicing. When compared to their linear mRNA counterparts, circRNAs exhibit greater stability due to their looped structure, which shields them from degradation by exonucleases. Although the majority of endogenous circRNAs do not serve as templates for protein synthesis, a few have been identified that can direct the production of proteins.^9^, ^10^ Despite the absence of the key components required for cap‐dependent translation, circRNAs can be genetically modified to facilitate protein synthesis. This can be achieved by incorporating internal ribosome entry sites (IRES) or by adding m6A modifications upstream of the ORF. These modifications enable the translation machinery to initiate protein production from the circRNA template.^11^, ^12^ Therefore, given their exceptional stability and potential for encoding proteins,, circRNAs can be used as an excellent vector for immunogen production. Wei's team presented a circRNA‒RBD vaccine platform that provided strong protection against the SARS‐CoV‐2 variant in mice and rhesus monkeys. This study represents the first successful application of the circRNA‒RBD vaccine platform.^13^

In this study, we confirmed the stability and longevity of protein expression triggered by circRNA. Following this, we assessed the therapeutic potential of circRNA‐based vaccines targeting tumor neoantigens in mice with subcutaneous and orthotopic tumors, as well as their preventive capabilities against hepatocellular carcinoma (HCC) in a preventive model. Our findings indicate that these vaccines can stimulate a robust antitumor immune response and foster a sustained, tumor‐specific immune memory by augmenting the population of central memory T cells in the spleen. This work offers preliminary evidence supporting the efficacy of circRNA neoantigen vaccines in treating HCC.

## 2 RESULT

### 2.1 In vitro synthesis of circRNA with protein translation function

Neoantigen vaccines have shown antitumor immune responses in a variety of tumors.^5^ In our previous study, using the murine HCC cell line Hepa1‐6, we screened and confirmed that seven mutation‐coding peptides (Ptpn2_I383T, Mapk3_S284F, Samd91_K752M, Lmf1_F523V, Traf7_C403W, Dtnb_K40T, and Lbr_A341P) could elicit significant immune responses in mice. Among them, Ptpn2_I383T has higher immunogenicity.^14^ To further develop circular RNA‐based neoantigen vaccines, we selected the Ptpn2_I383T peptide (KRWLYWQPTLTKMGFVS [mutant amino acid underlined], namely PTPN2) as the target neoantigen.

**FIGURE 1:**
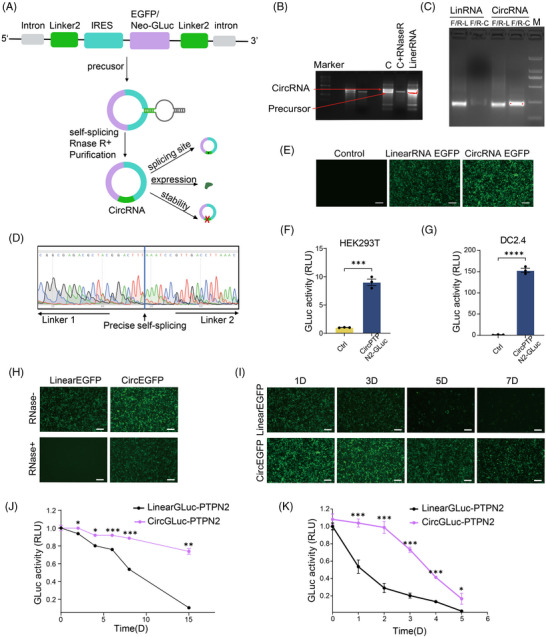
Protein expression initiated by circular RNA (circRNA). (A) Schematic diagram of circRNA circularization via the permuted intron‒exon (PIE) system. (B) Agarose gel confirmation of RNA circularization. (C) Precursor RNA subjected to circularization conditions. C + RNase R: digested with RNase R. (C) q‐PCR and (D) Sanger sequencing detect cyclic sites. (E) Expression of circRNA and linearRNA (linRNA) in HEK293T cell transfected with circRNA enhanced green fluorescent protein (EGFP) or linRNA EGFP. Scale bars: 400 µm. (F and G) Gaussian luciferase test showing the expression of PTPN2‐GLuc in HEK293T and DC2.4 cells. (H) The expression stability of circRNA compared to linear mRNA after RNase R treatment. (I and J) The stability of circRNA compared to linear mRNA were by observing the differences in the expression of GFP and GLuc after placed at room temperature for different times, respectively. Scale bars: 400 µm. (K) Translation efficacy of circRNA compared to linear mRNA after transfected different times. ^*^ *p* < 0.05, ^**^ *p* < 0.01, and ^***^ *p* < 0.001.

First, to construct an exogenous circRNA‐based protein expression system, we designed a self‐splicing intron to efficiently circularize the RNA, and a pair of homology arms and linkers was inserted at the specified location along the circRNA backbone.^11^ The IRES element was utilized to initiate translation (Figure 1A). We used enhanced green fluorescent protein (EGFP) or Gaussian princeps luciferase (Gluc) to detect the protein expression status of this system. First, RNA electrophoresis results indicated the formation of circRNA after the splicing reaction (Figure 1B). To ensure that the major splicing product was circular, we performed q‐PCR experiments with primers for linearRNA (linRNA) and primers for circRNA across the looping site, respectively (Figure 1C). The results showed that the primers of both linRNA and circular RNA generated PCR products. However, when linRNA was used as a template, circular RNA primers did not result in any PCR products. This confirms the presence of circRNA looping sites and verifies that the primary splicing outcome was circular. Sanger sequencing was performed, which demonstrated that the circRNA underwent self‐splicing and cyclization at the expected site (Figure 1D).

To confirm the protein expression of the circRNA system, we transfected gel‐purified circRNA into 293T cells and monitored protein expression efficiency using EGFP. Our findings indicate that circular RNA exhibits remarkably higher green fluorescence brightness than linRNA (Figure 1E). To investigate whether the circular RNA protein expression system can express the PTPN2 neoantigen polypeptide target, we fused the polypeptide‐encoding sequence with Gluc for expression. The activity of Gluc served as an indirect indicator of PTPN2 neoantigen polypeptide expression. The data demonstrated that Gluc activity significantly increased in cells transfected with the circular RNA system in both 293T and DC2.4 (Figure 1F,G). These findings suggest that neoantigen polypeptides can be expressed using this system.

circRNA exhibits exceptional stability, a characteristic attributed to its covalently closed loop configuration that shields it from degradation by exonucleases. To verify this advantage of circRNA neoantigen vaccines, we pre‐treated EGFP‐expressing circRNA and linRNA with RNase R (a 3′ exonuclease that only affects linRNAs^15^). As depicted in Figure 1H, the activity of EGFP was totally suppressed in the linRNA group upon treatment with RNase R, whereas in the circRNA group, there was a robust expression of EGFP. Resistance to nuclease‐mediated degradation of circRNA was also confirmed using fetal bovine serum (FBS)‐mediated degradation (Figure S1). The circRNA and linRNA were left at room temperature for varying periods, and subsequently, their ability to express EGFP was tested. The results showed a significant decrease in linRNA's ability to express EGFP over time, with minimal expression observed on the 7th day. In contrast, circRNA demonstrated robust stability and continued to express EGFP efficiently even after 7 days (Figure 1I). Similar results were attained when the GLuc protein replaced the targeted protein (Figure 1J). These results indicated that linRNA‐expressed proteins' efficiency diminished considerably with increased room temperature storage time, while circRNA's protein expression efficiency remained relatively constant. In addition, circRNA showed a half‐life of protein production of about 88 h, while the half‐life of linRNA protein production was approximately 27 h (Figure 1K). These data suggest that the circRNA protein expression system is more stable and longer lasting than that of linRNA.

### 2.2 circRNA vaccine induces immune response in vitro

**FIGURE 2:**
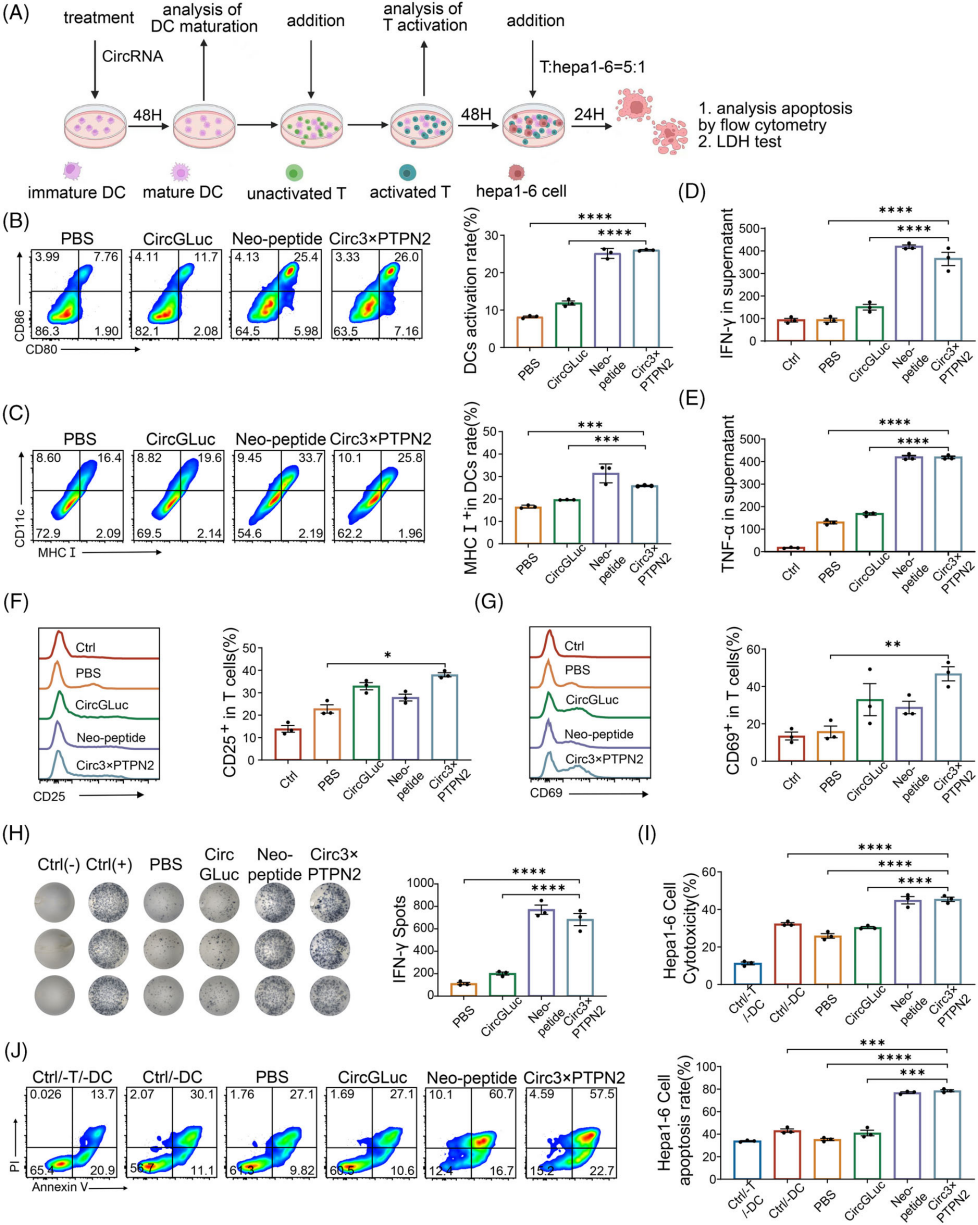
Immunogenicity validation of circular RNA (circRNA) vaccine in vitro. (A) Overview of in vitro immunogenicity validation. (B and C) Flow cytometry analysis of the percentage of CD80, CD86, major histocompatibility complex (MHC) I in dendritic cells (DCs) after treated with PBS, circRNAGluc, Neo‐Polypeptide, and circRNA3×PTPN2, the right panel shows the statistical analysis of the data. (D and E) Detection of interferon‐gamma (IFN‐γ) and tumor necrosis factor‐alpha (TNF‐α) secretion in culture medium by ELISA. (F and G) Flow cytometry analysis of the percentage of active T cell by tests CD25 and CD69, the right panel shows the statistical analysis of the data. (H) IFN‐γ spot‐forming cells and statistical data from stimulated T cell of PBS, circRNAGluc, Neo‐Polypeptide, and circRNA3×PTPN2 via ELISPOT assay. (I) LDH test the cytotoxicity of Hepa1‐6 cells by T cells. (J) Flow cytometry analysis the apoptosis rate of Hepa1‐6 cell after treated with activated T cells, the right panel shows the statistical analysis of the data. ^*^ *p* < 0.05, ^**^ *p* < 0.01, ^***^ *p* < 0.001, and ^****^ *p* < 0.0001. LDH, lactate dehydrogenase; PBS, phosphate buffered saline.

To assess the in vitro immune response of the circRNA neoantigen vaccine, we evaluated its impact on DC maturation, T‐cell activation, and T‐cell‐induced tumor cell killing through in vitro experiments (Figure 2A). To enhance the expression of polypeptide fragments, we incorporated a repeat sequence of three polypeptide fragments 3×PTPN2. Peptide vaccine (Neo‐Polypeptide) and circRNA expressing Gluc alone (circRNA Gluc) were used as the positive and negative controls, respectively. The experimental results demonstrated that the proportion of CD80+CD86+DCs in the circRNA3×PTPN2 group was 26.0% ± 0.12%, which was much higher than that in the phosphate buffered saline (PBS) (8.17% ± 0.21%) or circRNAGluc (11.87% ± 0.55%) groups (Figure 2B). In addition, circRNA3×PTPN2 also significantly increased the expression of major histocompatibility complex (MHC) I in DCs (Figure 2C). This indicates that the circRNA neoantigen vaccine can significantly promote DC activation.

To examine the effect of the circRNA neoantigen vaccine on T‐cell activation, DCs were exposed to the neoantigen and then co‐cultured with T cells. Flow cytometry was used to identify any changes in the expression of T‐cell activation markers CD25 and CD69. The data showed that these matured DCs induced by circRNA3×PTPN2 activated a higher percentage of CD25+T (38.25% ± 1.55%) or CD69+T (46.73% ± 3.78%) cells compared to PBS treatment (23.75% ± 2.75% and 15.67% ± 3.13%) (Figure 2D,E). T‐cell activation by the circRNA neoantigen vaccine was also confirmed by cytokine secretion of interferon‐gamma (IFN‐γ) and tumor necrosis factor‐alpha (TNF‐α) (Figure 2F,G). An ex vivo IFN‐γ ELISPOT assay demonstrated that mice treated with the circRNA neoantigen exhibited efficient IFN‐γ secretion and T‐cell activation in vivo (Figure 2H). Additionally, the circRNA neoantigen vaccine significantly enhanced T‐cell killing of Hepa1‐6 tumor cells when compared to the PBS or circRNA‐Gluc group, as shown by flow cytometry and lactate dehydrogenase (LDH) assay (Figure 2I,J). These results indicate that circRNA neoantigens can induce DC‐cell maturation and T‐cell activation. This cellular immune effect can further promote T‐cell killing of tumor cells.

### 2.3 Characterization of the circRNA‒lipid nanoparticle complex: in vitro and in vivo studies

**FIGURE 3:**
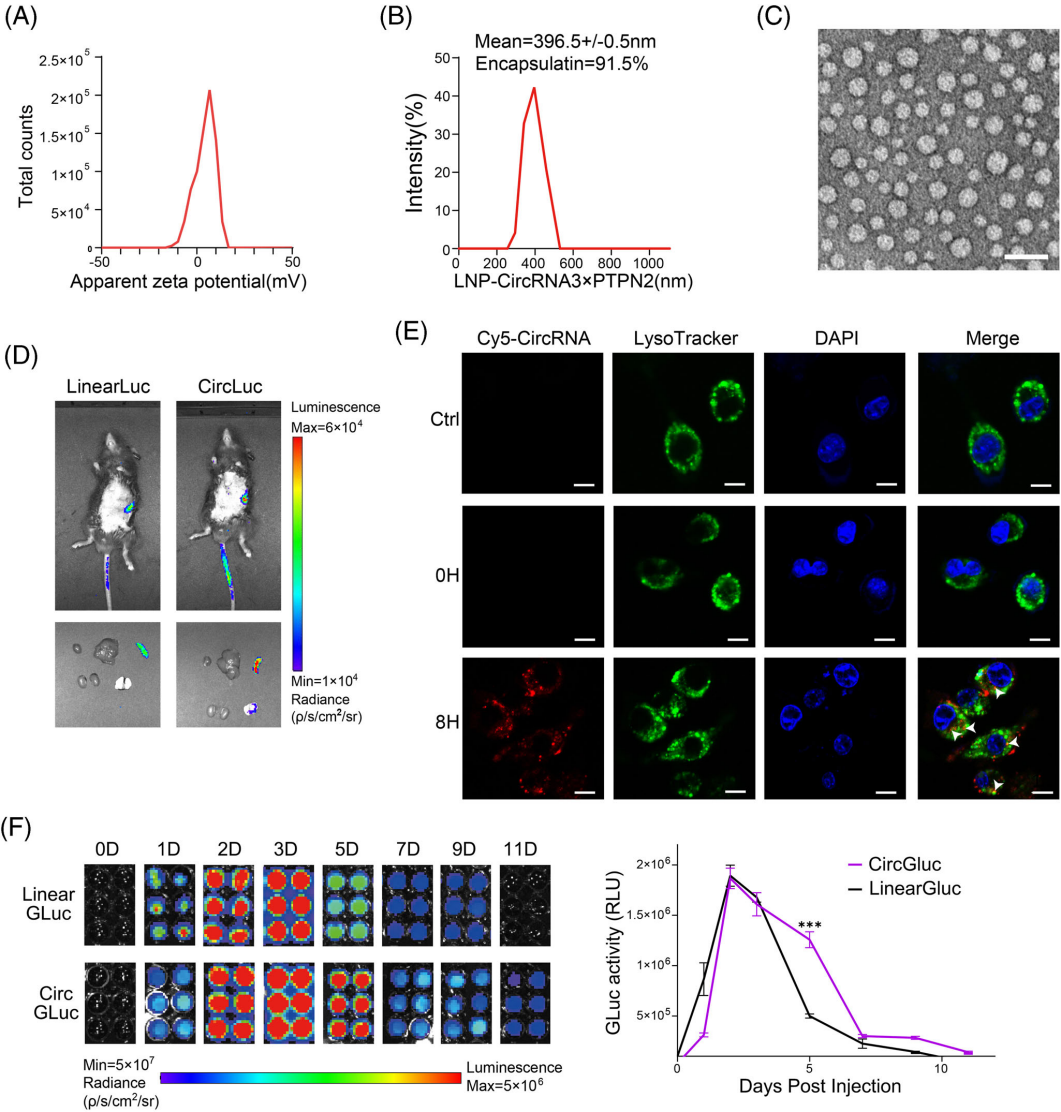
Characterization of the circular RNA (circRNA)‒lipid nanoparticle (LNP) complex. (A) Zeta potential of the circRNA3×PTPN2 complex (pH 7.4). (B) Encapsulation of circRNA3×PTPN2 and size distributions of the circRNA3×PTPN2 complex. (C) Transmission electron microscopy (TEM) image of the circRNA3×PTPN2‒LNP complex. Scale bar: 100 nm. (D) Bioluminescence images of mouse after injected the circRNALuc, circRNALuc were given tail vein to C57/B6 mice (10 µg RNA per mouse), luciferase expression was measured 12 h post‐injection. (E) Intracellular localization of the Cy5‐labeled circRNA characterized by Confocal Laser Scanning Microscope. Scale bar: 20 µm. (F) The stability of circRNAs was analyzed by biofluorescence imaging of Gaussian luciferase expression in peripheral blood serum of mice at different times after injection of circRNAGluc. circRNAGluc and linRNAGluc were injected into the tail vein of C57/B6 mice (10 µg of RNA per mouse). ^***^ *p* < 0.001.

Lipid nanoparticles (LNPs) have demonstrated the ability to effectively encapsulate and deliver circRNA molecules, promoting their escape from the endosome and enabling their translation in vivo.^16^ In our study, we harnessed LNPs to administer a circRNA vaccine encoding for 3×PTPN2 (referred to as circRNA3×PTPN2) to explore its in vivo functionality. After purification, the circRNA was successfully encapsulated within LNPs, resulting in a stable complex. The zeta potential for the LNP‒circRNA3×PTPN2 complex ranged from 0 to 10 mV, as illustrated in Figure 3A. The encapsulation efficiency of the LNPs reached 91.5%, with the LNP‒circRNA3×PTPN2 complex exhibiting a mean diameter of 396.5 ± 0.5 nm, as shown in Figure 3B. Additionally, the morphology of the circRNA‐loaded LNPs was examined using scanning electron microscopy, and the results are presented in Figure 3C.

In vivo delivery was monitored using IVIS bioluminescence imaging, and the results showed that LNP mainly targeted the circRNA vaccine to the spleen (Figure 3D). The intracellular trafficking of the LNP‒circRNA3×PTPN2 complex was examined using confocal microscopy by labeling the complex with the fluorescent dye Cy5 (Figure 3E). The images showed that most of the complex entered the cell and partly localized within the lysosome after 8 h of incubation. To verify the duration of circRNA protein expression in vivo, we injected GLuc‐expressing circRNA or linRNA into the mice via the tail vein and measured GLuc activity in mouse serum after varying time points. Our findings indicate that circRNA‐expressed protein could endure for more than 11 days, whereas linRNA could only persist for 9 days. On the 5th day, the quantity of protein expressed by linRNA significantly decreased, whereas circRNA maintained a high level of protein expression (Figure 3F). Hence, the above findings suggest that the LNP‐based delivery system is suitable for in vivo administration of circRNA vaccine. Moreover, the protein's stability, which is expressed by circRNA, is greater than that of linRNA.

### 2.4 Therapeutic efficacy of circRNA vaccine in subcutaneous murine HCC tumor model

**FIGURE 4:**
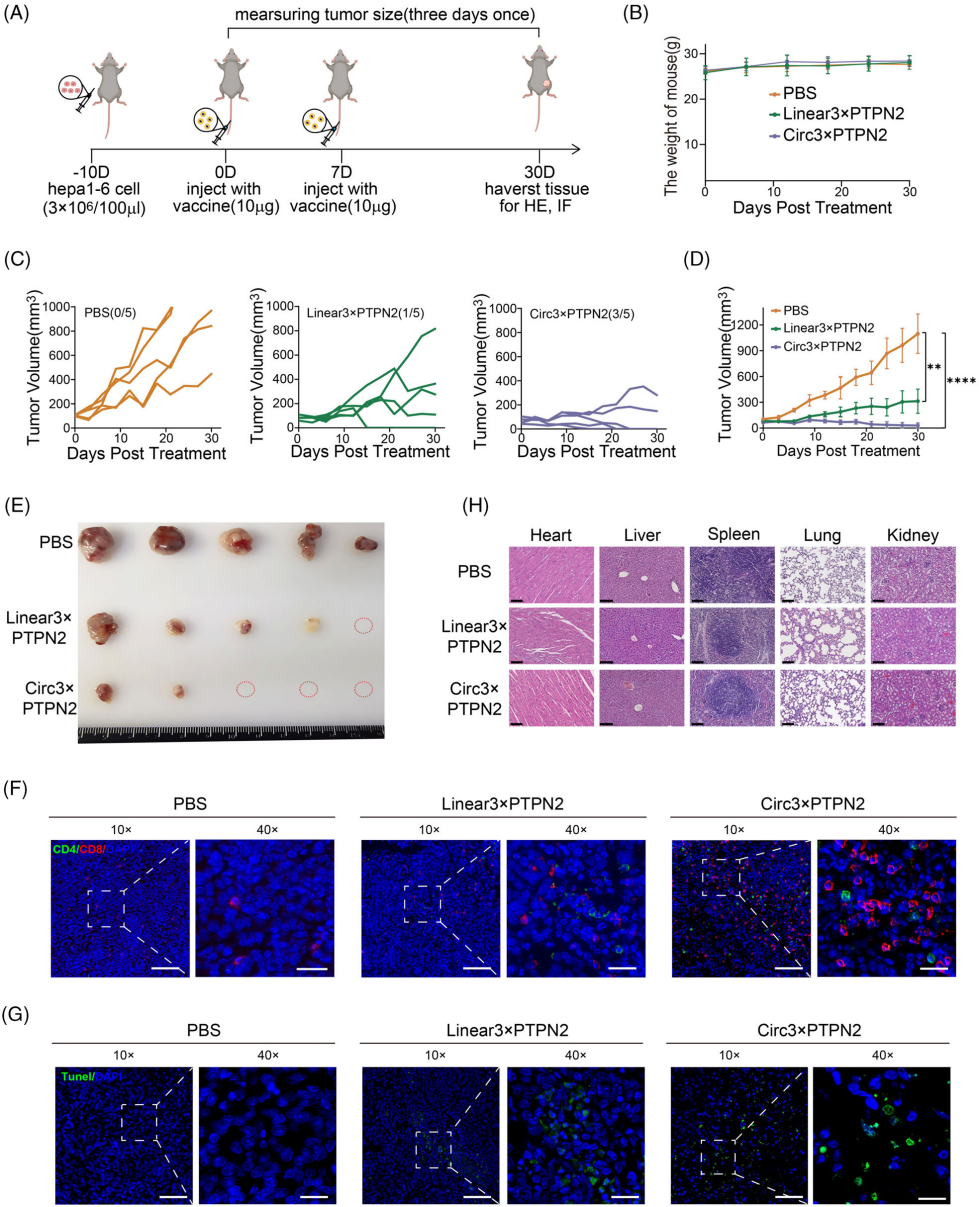
Anti‐tumor efficacy of circular RNA (circRNA)‒lipid nanoparticle (LNP) vaccine in subcutaneous hepatocellular carcinoma (HCC) model. (A) Treatment timeline of the experiment to evaluate the anti‐tumor efficacy. (B) Body weight curves of mice during treatment. (C) Tumor growth curves of each group. (D) Average tumor growth curve of mice after treat with PBS, linRNA3×PTPN2, circRNA3×PTPN2 (*n* = 5). (E) Images of tumor size. (F) The representative immunofluorescence image of CD4^+^ and CD8^+^ T‐cell infiltration in tumor tissues. Scale bars: 20 and 100 µm. (G) The representative immunofluorescence image of terminal‐deoxynucleotidyl transferase‐mediated nick end labeling (TUNEL) in tumor tissues. Scale bars: 20 and 100 µm. (H) Hematoxylin and eosin (H&E) staining for vital organs in mice. Scale bars: 100 µm. ^**^ *p* < 0.01 ^****^ *p* < 0.0001. PBS, phosphate buffered saline.

To further verify the anti‐tumor immune effect of the circRNA vaccine in vivo, we first established a subcutaneous Hepa1‐6 tumor model. Tumor‐bearing mice were injected twice with LNP‒circRNA3×PTPN2 via the tail vein (Figure 4A). As shown in Figure 4B, circRNA vaccination did not affect the weight of the mice. However, both linRNA vaccine and circRNA vaccine can significantly inhibit tumor growth. At day 30, circRNA vaccination displayed the highest efficiency in suppressing tumors when compared to PBS (0/5, 0%) and linRNA (1/5, 20%). Notably, 60% (3/5) of tumors completely disappeared (Figure 4C‒E). To verify whether the antitumor effect of circRNA neoantigens is dose dependent, we injected mice with varying doses of the circRNA vaccine. The results showed that the circRNA neoantigen vaccine inhibited tumor growth in a dose‐dependent manner when compared to the circRNA vector control (Figure S2).

Neoantigens are presented by antigen‐presenting cells and subsequently recognized by T cells, which triggers an anti‐cancer immune response in patients. First, we evaluated vaccine‐induced T‐cell immunity in the tumor region. Mice were sacrificed and tumor tissue sections were prepared for immunofluorescence staining. The results indicated that the circRNA3×PTPN2 group had a significantly higher number of T‐cell infiltration in tumor tissues compared to the PBS group and linRNA group (Figure 4F). The circRNA vaccine was shown to significantly induce tumor cell apoptosis by terminal‐deoxynucleotidyl transferase‐mediated nick end labeling staining of tumors in situ (Figure 4G).

Hematoxylin and eosin (H&E) staining of major organs was used to evaluate the systemic toxicity of the vaccine. No obvious toxic side effects were observed (Figure 4H). To investigate the impact of the circRNA vaccine on the liver function of mice, we examined mouse plasma liver function indicators. The results showed that the circRNA vaccine had no significant effect on mouse liver function (Figure S3).

Therefore, the above results indicate that the circRNA neoantigen vaccine we constructed can exert anti‐tumor immune effect and inhibit tumor growth in the mouse subcutaneous tumor model without any detectable side effects.

### 2.5 Tumor therapeutic efficacy of circRNA vaccine in orthotopic HCC models

**FIGURE 5:**
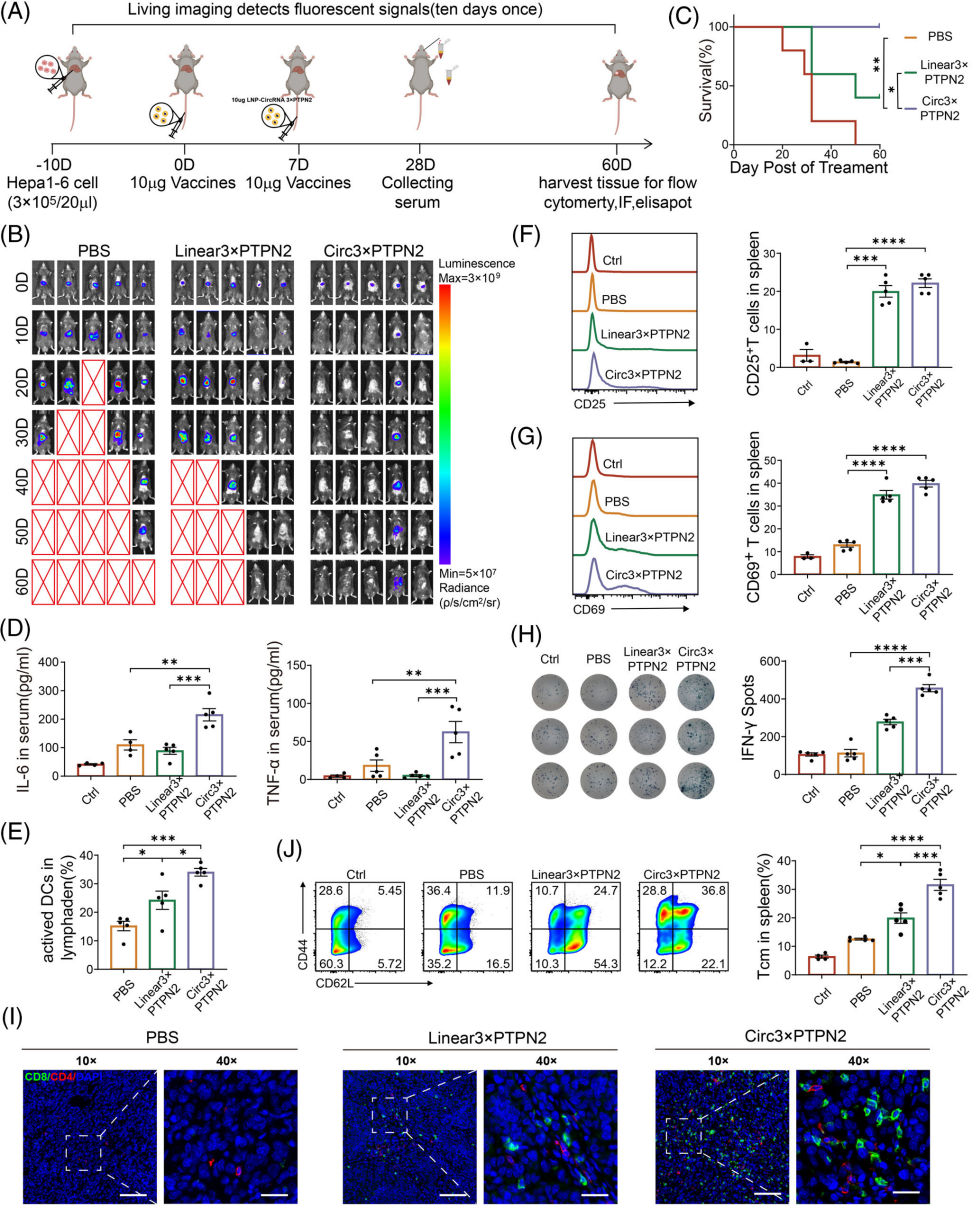
Anti‐tumor efficacy of circular RNA (circRNA)‒lipid nanoparticle (LNP) vaccine in orthotopic hepatocellular carcinoma (HCC) model. (A) Treatment timeline of the experiment to evaluate the anti‐tumor efficacy. (B) Tumor burden monitoring of PBS, linRNA3×PTPN2, and circRNA3×PTPN2‐treated mice by bioluminescence imaging. (C) Kaplan‒Meier survival curves of PBS, linRNA3×PTPN2, and circRNA3×PTPN2‐treated groups. (D) Serum cytokine interleukin‐6 (IL‐6) and tumor necrosis factor‐alpha (TNF‐α) release after circRNA3×PTPN2 administration via ELISA assays. (E) Flow cytometry analysis the percentage of matured dendritic cells (DCs) in lymph nodes after different treatment. (F and G) Flow cytometry analysis the percentage of CD25+ and CD69+ T cells in spleen after different treatment. (H) Interferon‐gamma (IFN‐γ) spot‐forming cells and statistical data from stimulated T cell of PBS, circRNAGluc, Neo‐Polypeptide, and circRNA3×PTPN2 via ELISPOT assay. (I) The representative immunofluorescence image of CD4+ and CD8+ T‐cell infiltration in tumor tissues. Scale bar: 20 µm. (J) Flow cytometry analysis the percentage of central memory T cells in spleen after different treatment and the statistical analysis. ^*^ *p* < 0.05, ^**^ *p* < 0.01, ^***^ *p* < 0.001, and ^****^ *p* < 0.0001. PBS, phosphate buffered saline.

We further investigated the function of the circRNA vaccine in an in situ tumor model (Figure 5A). An intrahepatic tumor model with Hepa1‐6 cells expressing luciferase was successfully established. Subsequently, mice bearing orthotopic tumors were randomly assigned to one of three groups for treatment: a PBS control group, a group receiving linRNA, and a group treated with circRNA. Fortunately, compared with the PBS and linRNA treatment groups, the circRNA treatment group significantly reduced the luciferase activity at the tumor site with the extension of treatment time, due to the excellent therapeutic effects. After vaccination, 80% (4/5) of tumors were entirely eradicated among the circRNA vaccination group, whereas the PBS group (0/5, 0%) and linRNA group (2/5, 40%) exhibited significantly lower reduction rates (Figure 5B). Excitingly, we also found that mice receiving the circRNA vaccine prolonged the overall survival time of mice compared with the PBS or linRNA group (Figure 5C). Peripheral blood samples were obtained for an ELISA to measure pro‐inflammatory cytokines. The innate immune response was characterized by the levels of serum IL‐6 and TNF‐α observed in the group administered the circRNA vaccine (Figure 5D).

To determine whether the innate immune response induced by the circRNA vaccine is due to the expression of neoantigens or the circRNA itself, a deletion mutation was constructed on the IRES of the circRNA (deletion of CVB3 domain II)^17^ (Figure S4A). The mutant vector was found to be incapable of expressing protein, as indicated by the Gluc activity assay (Figure S4B). In vitro experiments demonstrated that the mutant vector (Circ3×PTPN2‐IRES mut) did not induce an increase in TNFα and IFN‐γ (Figure S4C,D). This suggests that the circRNA neoantigen vaccine elicits an innate immune response through the neoantigen polypeptide it expresses, rather than through the circRNA itself.

To ascertain the impact of circRNA vaccine injection on DC activation and T‐cell immunity, we initially assessed the activation status of mouse lymph node DC. It was observed that there was a higher proportion of mature DCs in the circRNA vaccine group compared to the PBS (*p* < 0.001) and linRNA (*p* < 0.05) groups (Figure 5E). Second, we analyzed effector T cells within the spleen of mice that received various treatments using flow cytometry. The analysis revealed that the circRNA vaccine resulted in a significant increase in the percentage of CD25+ and CD69+ T cells when compared to the PBS group (*p* < 0.0001) (Figure 5F,G).

ELISPOT analysis was conducted to evaluate the neoantigen‐specific reactivity of splenic T cells against a panel of neoantigens. The results demonstrated that mice treated with the circRNA vaccine exhibited a significantly higher number of IFN‐γ spots (Figure 5H). Immunofluorescence staining of tumor tissue sections showed a significant increase in tumor‐infiltrating T lymphocytes in the circRNA vaccine treatment group (Figure 5I).

Moreover, analysis of the splenic T‐cell subpopulation revealed that treatment with the circRNA vaccine significantly augmented the proportion of CD44+CD62L+ central memory T cells in the spleen when compared to the PBS (*p* < 0.0001) or linRNA vaccine group (*p* < 0.001) (Figure 5J). This indicates that the circRNA vaccine could trigger an enduring memory immune response to deter tumor recurrence.

Therefore, the circRNA neoantigen vaccine exerts anti‐tumor immune effects in the subcutaneous murine HCC tumor model by inducing both innate and adoptive immune responses.

### 2.6 Prophylactic effects of circRNA vaccine in Hepa1‐6 tumor‐bearing mice

**FIGURE 6:**
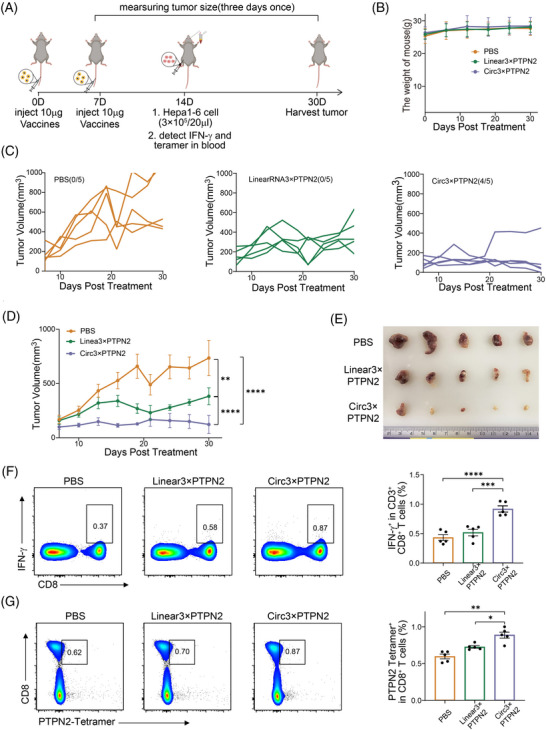
Anti‐tumor efficacy of circular RNA (circRNA)‒lipid nanoparticle (LNP) vaccine in prophylactic hepatocellular carcinoma (HCC) model. (A) Schematic diagram showing the timeline of establishing prophylactic HCC model. (B) Body weight curves of mice during treatment. (C) Tumor growth curves of each group and (D) average tumor growth curve of mice after treat with PBS, linRNA3×PTPN2, circRNA3×PTPN2 (*n* = 5). (E) Images of tumor size. (F and G) Flow cytometry analysis showed the percentage of PTPN2 tetramers and INF‐γ specific CD8+ T cells in infiltrating CD8+ T cells (*n* = 5). ^*^ *p* < 0.05, ^**^ *p* < 0.01, ^***^ *p* < 0.001, and ^****^ *p* < 0.0001. PBS, phosphate buffered saline.

To evaluate the tumor preventive capacity of the circRNA vaccine, C57BL/6 mice were injected twice with the vaccine through the tail vein 7 days apart. Hepa1‐6 cancer cells were injected subcutaneously 7 days later (Figure 6A). Vaccination did not affect the weight of the mice (Figure 6B). In mice inoculated with PBS, tumor growth was rapid, whereas in mice inoculated with the vaccine, tumor growth was significantly delayed. More importantly, the circRNA vaccine had a better effect on tumor inhibition compared to linRNA (Figure 6C‒E). Interestingly, the proportion of IFN‐γ+ CD8+ T cells was significantly upregulated in the circRNA vaccine treatment group, indicating that the circRNA vaccine could enhance antigen‐specific IFN‐γ CD8+ T‐cell responses (Figure 6F). Peptide‐major histocompatibility complex (pMHC) tetramer staining indicated that CD8+ T cells specific for Ptpn2 were detectable in the peripheral blood mononuclear cells (PBMCs) of mice that received the circRNA vaccine (Figure 6G). These results further demonstrate that circRNA vaccines induce anti‐tumor immunity by inducing innate and adoptive immune responses and prevent tumor development.

## 3 DISCUSSION

In vivo, circRNAs are usually produced by reverse splicing of RNA. In vitro, chemical and enzymatic protocols can be used to generate circular RNA, but these methods are more suitable for small and medium‐sized RNAs. The circularization of larger RNA molecules presents a significant challenge, particularly when it comes to modified group I introns that have undergone a permutation of introns and exons, a technique known as the permuted intron‒exon (PIE) strategy. This approach has demonstrated considerable potential.^18^ In our study, the PIE strategy was used to generate circRNAs, an approach that allowed us to generate circRNAs of sufficient length to insert efficient IRES elements and antigen coding sequences. We used this strategy to establish a circRNA protein expression system. Compared to linRNA, the circRNA protein expression system is more stable. Because of the enhanced stability of circRNA, the expression levels of antigens are more elevated and sustained compared to those produced by mRNA vaccines, and finally showed better anti‐tumor immune effects in animal experiments.

The effectiveness of a drug or vaccine is significantly influenced by its delivery system. Various targeted delivery systems have been developed to enhance drug efficacy.^19^, ^20^, ^21^ Lipid nanoparticles (LNPs) represent a prevalent system for delivering RNA. When LNPs are used to encapsulate circRNA in vitro, the resulting complexes can be internalized by cells via endocytosis upon administration.^22^, ^23^ Upon cellular entry, the LNP complex can be broken down by enzymes from the lysosome. Similarly, circRNA can be degraded by lysosomal enzymes, which in turn facilitates the release of the encapsulated circRNA. This release then enables the expression of the encoded neoantigens. This process was also verified in our study (Figure 3E). Subsequently, neoantigens expressed by circRNA are captured and internalized by DCs, degraded by proteolysis, and presented to CD8+ and CD4+ T cells that initiate the adaptive immune response. Furthermore, circRNA that enters cells can stimulate the innate immune response by triggering the retinoic acid‐inducible gene‐I (RIG‐I) pathway, a process that has been demonstrated to enhance the anti‐tumor immune response.^24^, ^25^ Our study also showed that circRNA vaccines induce anti‐tumor immunity by inducing both innate and adoptive immune responses.

The immunogenicity of circRNA remains controversial. Research indicates that unmodified exogenous circRNA can successfully evade detection by cellular RNA sensors, thereby preventing the activation of immune responses in cells capable of recognizing RIG‐I and Toll‐like receptors, as well as in mice.^26^ However, another study showed that exogenous circRNAs have been demonstrated to be effective adjuvants, capable of inducing antigen‐specific T‐cell activation, antibody production, and anti‐tumor immunity in vivo. However, it has been shown that m6A modifications can negate the activation of immune genes and the adjuvant effects of circRNAs.^27^ Chen Lingling's team compared the immunogenicity of circRNAs synthesized by different methods in vitro and found that circRNAs synthesized by T4 RNA ligase, including circRNA aptamers and translatable circRNAs, did not induce intracellular natural immune responses. However, the circRNA formed by self‐splicing of two different type I introns, T4 bacteriophage and Anabeana, would introduce 74 and 186 foreign nucleotides, respectively, and thereby induce the intracellular natural immune response.^28^ The group I catalytic intron sequence from Anabaena used in our study is immunogenic and can play the role of a self‐adjuvant. In our in vivo experiments, circRNA induced higher expression of IL‐6 and TNF‐α than linRNA, which also helped to prove the immunogenicity of circRNA (Figure 5D).

Somatic mutations can generate neoantigens that are distinctive to cancer cells and not present in healthy tissues. This characteristic renders them highly desirable targets for the development of immunotherapeutic strategies. p53R175H, KRAS G12D, and other common mutations in tumors can induce anti‐tumor immune responses as neoantigens.^29^, ^30^ In our previous study, we found the I383T mutation in Ptpn2 in the murine HCC hepa1‐6 cell line. Peptides that encode for PTPN2 mutations have been shown to elicit substantial immune responses in immunized mice. This immune activation is achieved through the use of autologous splenic T cells, which are stimulated by each neoantigen peptide presented on autologous mature DCs. Importantly, this immune response does not exhibit cross‐reactivity with the corresponding wild‐type peptides.^14^ In this study, we used circRNA to encode neoantigen polypeptides. circRNA has higher immunogenicity compared to polypeptide vaccines and can induce more powerful innate immunity, making it a potent self‐adjuvant. Additionally, it can compensate for the deficiency of complex synthesis in vitro and the high cost of polypeptide vaccines. In addition, although ovalbumin peptide has been used as circRNA antigen in recent studies to explore its anti‐tumor immune effect,^16^, ^31^ and our study used the endogenous mutation sequence of tumor cells as circRNA neoantigen vaccine, which was the first true study of circRNA neoantigen vaccine, and the results provide a proof‐of‐concept demonstration of the efficacy of circRNA neoantigen vaccine in cancer treatment.

Taken together, we developed a circRNA neoantigen vaccine platform for cancer therapy. circRNA has demonstrated greater stability and more sustained protein expression compared to linRNA. Vaccination with circRNA has been shown to provoke a significant innate immune response and a robust antigen‐specific T‐cell reaction, displaying promising anti‐tumor efficacy across various tumor models. Due to its high stability and relatively simple design process, the circRNA vaccine is expected to be an attractive vaccination alternative with great potential for clinical translation.

## 4 MATERIALS AND METHODS

### 4.1 Plasmid construction

The circRNA backbone, T7 promoter, homology arm, group I intron sequence, IRES sequence, linker sequence, and elements from a PIE construct were synthesized by General Biol and then cloned into the pET‐28a (+) plasmid. The encoding sequence for neoantigen PTPN2, EGFP, GLuc, and luciferase, were also synthesized by General Biol and cloned into the same pET‐28a (+) plasmid for use in the following IVT reaction. Further details on the plasmid sequence can be found in Table S1.

### 4.2 Production and purification of RNA

circRNA was produced following a previously reported method.^11^ The constructs were inserted into the pET‐28a (+) plasmid and then transferred into *Escherichia coli*. The circRNA vector fragments were recovered through agarose gel electrophoresis. The circRNA was transcribed using the T7 High Yield RNA Transcription Kit (New England Biolabs [NEB]) in accordance with the manufacturer's instructions. Following IVT, the reaction was treated with DNase I (NEB) for 20 min at 37°C. Subsequently, the linearized circRNA was purified using a column from the MEGAclear Transcription Purification Kit (Ambion). The mass and concentration of the purified product were then determined to ensure quality. For cyclization, GTP was added to a final concentration of 10 mM after column purification. The reaction was then heated at 55°C for 15 min and immediately placed on ice. To achieve a higher degree of circRNA purification, we supplemented the reaction with 20 U of RNase R and 5 µL of a 10‐fold concentrated RNase R buffer from Geneseed. Following this, the reaction was maintained at a temperature of 37°C for a period of 15 min. Midway through the incubation, an extra 10 U of RNase R was introduced to the mixture to enhance the purification process. The products were purified using the MEGAclear Transcriptional Purification Kit, and we determined the quality and concentration of the purified products using agarose gel electrophoresis. To confirm that circRNA was generated through a splicing reaction, RNA samples were digested with RNase R (Geneseed) for 15 min at 37°C, followed by agarose gel electrophoresis. The RNA samples were also reverse transcribed to cDNA and amplified by PCR using bridge primers. The samples were then sent for Sanger sequencing. To synthesize cy5‐labeled circRNA, cy5‐UTP was used instead of 25% UTP during IVT, followed by the same steps as in the appeal (Apex Bio).

linRNA was produced following the manufacturer's instructions. Briefly, the constructs were inserted into the pET‐28a (+) plasmid and then transferred into *E. coli*. The linRNA vector fragments were recovered through agarose gel electrophoresis. Subsequently, linRNA was transcribed using the Hiscribe T7 mRNA Kit with CleanCap Reagent AG (NEB) in accordance with the manufacturer's protocol. To refine the process of linRNA purification, we first treated the unmodified linear mRNA with DNase I to remove any residual DNA, utilizing a MEGAclear Transcription Clean‐up Kit from Ambion. Subsequently, the RNA was subjected to a thermal treatment by heating to 70°C for a period of 5 min, followed by rapid cooling on ice for 3 min. Once cooled, the RNA was capped using a combination of mRNA cap 2′‐O‐methyltransferase and vaccinia capping enzyme, both sourced from NEB, in strict adherence to the manufacturer's guidelines. The capped linear transcripts were modified by adding polyadenosine tails using *E. coli* PolyA polymerase (NEB) in accordance with the manufacturer's instructions. The fully processed mRNA was then purified using a column.

### 4.3 Mouse and cell lines

Male C57BL/6 mice (6‒8 weeks old) were obtained from Shanghai Slake Experimental Animals Co. Ltd. All mice were housed under specific pathogen‐free‐grade conditions in the animal facility of Fujian Medical University Mengchao Hepatobiliary Hospital. HEK293T cell line was purchased from ATCC. The DC2.4 cell line was purchased from ATCC. Hepa1‐6 cell lines were maintained in our laboratory; these cell lines were cultured in high glucose (Thermo) with 10% FBS (Excell) at 37°C with 5% carbon dioxide.

### 4.4 Cell transfection

For RNA transfection into HEK293T or DC2.4 cells, 5 × 10^5^ cells per well were seeded in six‐well plates. Three micrograms of RNA was transfected into cells using TransIT‐Mrna reagent (Mirus) according to the manufacturer's instructions. Twenty‐four hours after transfection, cells transfected with RNA‐EGFP were observed by fluorescence microscopy. The supernatants of cells transfected with RNA‐GLuc were collected and expression was detected by the Secrete‐Pair Gaussian Luciferase Assay (GeneCopoeia) according to the manufacturer's instructions.

### 4.5 Cell isolation

For bone marrow DCs, healthy male C57BL/6 mice were immersed in ethanol for 15 min, then the surrounding muscle tissue was removed with sterile gauze. Both ends of the bones were cut off and the bone marrow was rinsed three times with PBS. Then red blood cell lysate (Solarbio) was added to remove red blood cells. The remaining cells were cultured in 1640 medium supplemented with 20 ng/mL granulocyte‐macrophage colony‐stimulating factor and 10 ng/mL IL‐4. On day 3, half of the culture supernatant was replaced with the medium described above. On days 7−9, cells can be determined by flow cytometry and used for further experiments. For spleen T cells, the healthy male C57BL/6 mice were immersed in ethanol for 15 min, then the spleens were harvested and the cells in the spleens were collected, after which the T cells were isolated by Ficoll (TBD), the cells can be used for further experiments or cryopreservation.

### 4.6 Activation of DCs in vitro

DCs were seeded into six‐well plates at a density of 5 × 10^5^ cells/well, followed by transfection with different formulations for 48 h. Then, the cells were harvested and stained with anti‐CD11c‐APC (eBioscience), anti‐CD80‐PE (eBioscience), anti‐CD86‐PE‐Cy7 (eBioscience), and DC maturity was determined by flow cytometry.

### 4.7 Activation of T cells in vitro

DCs were treated with different formulations as described above, followed by seeding with 1 × 10^5^ extracted T cells and co‐culture for 24 h, then the cells were harvested and stained with anti‐CD3‐APC (eBioscience), anti‐CD69‐FITC (eBioscience), and anti‐CD25‐PECy5.5 (eBioscience) to detect the activity of T cells by flow cytometry, also the T cells were detected for the inflammatory factor IFN‐γ by ELISA pot assay. Briefly, the T cells were seeded at a density of 1 × 10^3^ cells/well in an ELISA pot plate, followed by DCs as described above were seeded at a ratio of 1:5 for co‐culture for 24 h. T cells were then detected for the inflammatory factor IFN‐γ according to the ELISA pot instructions (Mabtech) and the spots were analyzed using the ELISPOT analysis system (AT‐Spot‐2200).

### 4.8 Cytotoxic assay in vitro

Hepa1‐6 cells were seeded in a six‐well plate at a density of 3 × 10^6^, and then, 1 × 10^6^ T cells treated with different DCs as described above were seeded into the plate. After 48 h, the cells in the supernatant and Hepa1‐6 cells were collected, and then stained with anti‐CD3‐APC (eBioscience), Annexin V‐FITC, and PI (Transgen) to detect cell apoptosis by flow cytometry. The supernatant of the cell culture medium was collected and the cytotoxicity rate was detected by LDH Cytotoxicity Detection Kit (Takara); cytotoxicity rate = (exp. value ‒ low control)/(high control ‒ low control) × 100%; and the low control was Hepa1‐6 cell supernatant medium without any treatment. The high control was 1% Triton X‐100‐treated Hepa1‐6 cell supernatant medium.

### 4.9 Encapsulation of RNA by LNP

In order to encapsulate linRNA and circRNA, we used commercially available lipids (in vivo‐jetRNA). The in vivo‐jetRNA reagent was vortexed for 5 s and spun down before use. The diluted mRNA was homogenized by gently pipetting up and down after adding the in vivo‐jetRNA reagent at a ratio of 1:1 µg of mRNA to µL of in vivo‐jetRNA reagent. The solution was then incubated at room temperature for 15 min before performing injections in animals. The concentration and encapsidation rate of circRNA were measured using the Quant‐it RiboGreen RNA Assay Kit (Invitrogen). The LNP‒circRNA particle size was measured using a Malvern Zetasizer Nano‐ZS 300 (Malvern) through dynamic light scattering. Gene expression was analyzed between 6 and 72 h after injection.

### 4.10 In vivo RNA‒LNP vaccine injection assay

To detect circRNA delivery sites in vivo, LNP‒circRNALuc (10 µg/mouse) and LNP‒linRNALuc (10 µg/mouse) were injected via the tail vein. After 24 h, mice were injected intraperitoneally with D‐luciferin (PerkinElmer) and 5 min later bioluminescence was measured using the IVIS spectral imaging system.

### 4.11 Mouse tumor models

All animal experiments were approved by the Animal Ethics Committee of Mengchao Hepatobiliary Hospital of Fujian Medical University (MCHH‐AEC‐2023‐08).

For the subcutaneous tumor model, the C57BL/6 mouse was injected subcutaneously with 3 × 10^6^ Hepa1‐6 cells together with Matrigel (ABW) on day −10. When the mouse tumor volume had grown to approximately 100 mm^3^, the mice were randomly divided into three groups. circRNA3×PTPN2 (10 µg of circRNA per mouse), an equimolar amount of linRNA3×PTPN2 and PBS were injected into the tail vein of three parallel groups of mice on days 0 and 7 to add immunotherapy. Tumor volume was measured every 3 days. Tumor volume was calculated by formation: *V* = length × width × width/2 (mm^3^). The body weight of the mice was also weighed. Mice were harvested when the tumor volume had grown to approximately 1000 mm^3^, peripheral blood was collected to detect biochemical indicators and mouse tissues were collected for HE staining to assess circRNA‒LNP biosafety. Briefly, mouse tissues were collected, fixed, paraffin embedded, and sectioned, and heart, liver, spleen, lung, and kidney tissues were stained with H&E (Solarbio). The tumor tissues were sent to Sevier for immunofluorescence staining to detect the immune infiltration of CD8+ and CD4+ cells in the different treatment groups.

For orthotopic tumor model, the C57BL/6 mice were orthotopically injected with 3 × 10^5^/20 µL Hepa1‐6 cells on day −10. The tumor size of orthotopic liver tumor in mice was detected by small animal in vivo image (PerkinElmer) after 10 days and the mice were randomly divided into three groups. circRNA3×PTPN2 (10 µg circRNA per mouse), an equimolar amount of linRNA3×PTPN2 and PBS were injected into the tail vein of three parallel groups of mice on days 0 and 7 for immunotherapy. In vivo imaging was performed every 10 days to monitor tumor growth. Mice were harvested after 60 days. DCs were collected from the bone marrow to assess maturation by flow cytometry, and T cells were collected from the spleen to assess antigen‐specific T cells by flow cytometry and ELISPOT. Tumor tissues were also collected, fixed, paraffin‐embedded, sectioned, and then sent to Sevier for immunofluorescence staining to detect immune infiltration of CD8+ and CD4+ cells in different treatment groups. Peripheral blood was collected to detect the inflammatory factor IL‐6 and TNF‐α using the ELISA detection kit (Beyotime) according to the manufacturer's instructions.

For tetramer staining, PBMCs were Fc‐blocked with PBS containing 5% bovine serum albumin for 20 min on ice. They were then stained with 1 µL of anti‐mouse tetramer PE for epitopes (Ptpn2376‐384 (RWLYWQPTL):H‐2Kb, MBL) for 30 min at room temperature. After tetramer pre‐incubation, cells were stained with anti‐mouse CD8‐FITC mAb (eBioscience) for 20 min, rotating in the dark. Flow cytometry was performed on a flow cytometer (BD FACSVerse) and data were analyzed using FlowJo v.10.

For the prophylactic tumor model, circRNA3×PTPN2 (10 µg circRNA per mouse), an equimolar amount of linRNA3×PTPN2 and PBS was injected into the tail vein of three parallel groups of mice on days −7 and −14 to provide immune protection. Peripheral blood was collected on day 0 to detect the inflammatory factor IL‐6 and TNF‐α. On day 1, 3 × 10^6^ Hepa1‐6 cells were injected intravenously together with Matrigel to simulate tumor growth. The tumor volume was measured every 3 days. Changes in tumor volume were recorded and the body weight of the mice was also weighed. The mice were harvested when the tumor volume had grown to approximately 1000 mm^3^.

### 4.12 Statistical analysis

All data in this study were meticulously analyzed using GraphPad Prism and are presented as mean ± S.D. Unpaired *t*‐test with Welch's correction was used for comparison between two groups. Kaplan‒Meier analysis was applied for survival curves. The significance level was defined as ^*^ *p* < 0.05, ^**^ *p* < 0.01, ^***^ *p* < 0.001, and ^****^ *p* < 0.0001.

## AUTHOR CONTRIBUTIONS

Bixing Zhao and Xiaolong Liu conceptualized the study. Fei Wang, Guang Cai, Yingying Li, Shaodong Gao, and Fang Li performed experiments. Fei Wang, Yingchao Wang, Qiuyu Zhuang, Zhixiong Cai, Cuilin Zhang, Bixing Zhao, and Xiaolong Liu performed statistical analysis. Bixing Zhao and Xiaolong Liu prepared the manuscript. All authors have read and approved the final manuscript.

## CONFLICT OF INTEREST STATMENT

The authors declare they have no conflicts of interest.

## ETHICS STATEMENT

The study received special approval from the Animal Ethics Committee of Mengchao Hepatobiliary Hospital of Fujian Medical University (MCHH‐AEC‐2023‐08). All the applied procedures followed the Guidelines for the welfare and use of animals in cancer research.^32^ For an animal carrying a single tumor, the mean diameter should not normally exceed 1.5 cm in mice for therapeutic studies. Necessity and rationality statement: in the xenograft models utilized in this study, the irregular shape of the tumors resulted in some reaching a maximum diameter of over 1.5 cm. However, the mean diameter did not exceed 1.5 cm, the total tumor volume remained below 2000 mm^3^, and the tumor burden was kept to less than 5% of the host animal's standard body weight. After discussion and review by the Animal Ethics Committee, all tumor burdens in this study did not exceed the permission of the Animal Ethics Committee and were considered to be in accordance with ethical standards.

## Supporting information

Supporting Information

## Data Availability

The data that support the findings of this study are available from the corresponding author upon reasonable request.

## References

[1] Pastor F , Berraondo P , Etxeberria I , et al. An RNA toolbox for cancer immunotherapy. Nat Rev Drug Discov. 2018;17(10):751‐767. 30190565 10.1038/nrd.2018.132

[2] Chu Y , Liu Q , Wei J , Liu B . Personalized cancer neoantigen vaccines come of age. Theranostics. 2018;8(15):4238‐4246. 30128050 10.7150/thno.24387 PMC6096398

[3] Xie N , Shen G , Gao W , Huang Z , Huang C , Fu L . Neoantigens: promising targets for cancer therapy. Signal Transduct Target Ther. 2023;8(1):9. 36604431 10.1038/s41392-022-01270-x PMC9816309

[4] Blass E , Ott PA . Advances in the development of personalized neoantigen‐based therapeutic cancer vaccines. Nat Rev Clin Oncol. 2021;18(4):215‐229. 33473220 10.1038/s41571-020-00460-2 PMC7816749

[5] Peng M , Mo Y , Wang Y , et al. Neoantigen vaccine: an emerging tumor immunotherapy. Mol Cancer. 2019;18(1):128. 31443694 10.1186/s12943-019-1055-6 PMC6708248

[6] He Q , Gao H , Tan D , Zhang H , Wang JZ . mRNA cancer vaccines: advances, trends and challenges. Acta Pharm Sin B. 2022;12(7):2969‐2989. 35345451 10.1016/j.apsb.2022.03.011 PMC8942458

[7] Esprit A , de Mey W , Bahadur Shahi R , Thielemans K , Franceschini L , Breckpot K . Neo‐antigen mRNA vaccines. Vaccines (Basel). 2020;8(4):776. 33353155 10.3390/vaccines8040776 PMC7766040

[8] Rojas LA , Sethna Z , Soares KC , et al. Personalized RNA neoantigen vaccines stimulate T cells in pancreatic cancer. Nature. 2023;618(7963):144‐150. 37165196 10.1038/s41586-023-06063-y PMC10171177

[9] Gao X , Xia X , Li F , et al. Circular RNA‐encoded oncogenic E‐cadherin variant promotes glioblastoma tumorigenicity through activation of EGFR‐STAT3 signalling. Nat Cell Biol. 2021;23(3):278‐291. 33664496 10.1038/s41556-021-00639-4

[10] Zhang M , Zhao K , Xu X , et al. A peptide encoded by circular form of LINC‐PINT suppresses oncogenic transcriptional elongation in glioblastoma. Nat Commun. 2018;9(1):4475. 30367041 10.1038/s41467-018-06862-2 PMC6203777

[11] Wesselhoeft RA , Kowalski PS , Anderson DG . Engineering circular RNA for potent and stable translation in eukaryotic cells. Nat Commun. 2018;9(1):2629. 29980667 10.1038/s41467-018-05096-6 PMC6035260

[12] Yang Y , Fan X , Mao M , et al. Extensive translation of circular RNAs driven by N(6)‐methyladenosine. Cell Res. 2017;27(5):626‐641. 28281539 10.1038/cr.2017.31 PMC5520850

[13] Qu L , Yi Z , Shen Y , et al. Circular RNA vaccines against SARS‐CoV‐2 and emerging variants. Cell. 2022;185(10):1728‐1744.e16. 10.1016/j.cell.2022.03.044 PMC8971115 35460644

[14] Chen H , Li Z , Qiu L , et al. Personalized neoantigen vaccine combined with PD‐1 blockade increases CD8(+) tissue‐resident memory T‐cell infiltration in preclinical hepatocellular carcinoma models. J Immunother Cancer. 2022;10(9):e004389. 36113894 10.1136/jitc-2021-004389 PMC9486396

[15] Memczak S , Jens M , Elefsinioti A , et al. Circular RNAs are a large class of animal RNAs with regulatory potency. Nature. 2013;495(7441):333‐338. 23446348 10.1038/nature11928

[16] Li H , Peng K , Yang K , et al. Circular RNA cancer vaccines drive immunity in hard‐to‐treat malignancies. Theranostics. 2022;12(14):6422‐6436. 36168634 10.7150/thno.77350 PMC9475446

[17] Chen R , Wang SK , Belk JA , et al. Engineering circular RNA for enhanced protein production. Nat Biotechnol. 2023;41(2):262‐272. 35851375 10.1038/s41587-022-01393-0 PMC9931579

[18] Petkovic S , Muller S . RNA circularization strategies in vivo and in vitro. Nucleic Acids Res. 2015;43(4):2454‐2465. 25662225 10.1093/nar/gkv045 PMC4344496

[19] Ma W , Yang Y , Zhu J , et al. Biomimetic nanoerythrosome‐coated aptamer‐DNA tetrahedron/maytansine conjugates: pH‐responsive and targeted cytotoxicity for HER2‐positive breast cancer. Adv Mater. 2022;34(46):e2109609. 35064993 10.1002/adma.202109609

[20] Zhang T , Zhou M , Xiao D , et al. Myelosuppression alleviation and hematopoietic regeneration by tetrahedral‐framework nucleic‐acid nanostructures functionalized with osteogenic growth peptide. Adv Sci (Weinh). 2022;9(27):e2202058. 35882625 10.1002/advs.202202058 PMC9507378

[21] Tian T , Zhang T , Shi S , Gao Y , Cai X , Lin Y . A dynamic DNA tetrahedron framework for active targeting. Nat Protoc. 2023;18(4):1028‐1055. 36670289 10.1038/s41596-022-00791-7

[22] Eygeris Y , Gupta M , Kim J , Sahay G . Chemistry of lipid nanoparticles for RNA delivery. Acc Chem Res. 2022;55(1):2‐12. 34850635 10.1021/acs.accounts.1c00544

[23] Zhang Y , Sun C , Wang C , Jankovic KE , Dong Y . Lipids and lipid derivatives for RNA delivery. Chem Rev. 2021;121(20):12181‐12277. 34279087 10.1021/acs.chemrev.1c00244 PMC10088400

[24] Ma Z , Shuai Y , Gao X , Wen X , Ji J . Circular RNAs in the tumour microenvironment. Mol Cancer. 2020;19(1):8. 31937318 10.1186/s12943-019-1113-0 PMC6958568

[25] Kiaie SH , Majidi Zolbanin N , Ahmadi A , et al. Recent advances in mRNA‒LNP therapeutics: immunological and pharmacological aspects. J Nanobiotechnol. 2022;20(1):276. 10.1186/s12951-022-01478-7 PMC9194786 35701851

[26] Wesselhoeft RA , Kowalski PS , Parker‐Hale FC , Huang Y , Bisaria N , Anderson DG . RNA circularization diminishes immunogenicity and can extend translation duration in vivo. Mol Cell. 2019;74(3):508‐520.e4. 10.1016/j.molcel.2019.02.015 PMC6724735 30902547

[27] Chen YG , Chen R , Ahmad S , et al. N6‐methyladenosine modification controls circular RNA immunity. Mol Cell. 2019;76(1):96‐109.e9. 10.1016/j.molcel.2019.07.016 PMC6778039 31474572

[28] Liu CX , Guo SK , Nan F , Xu YF , Yang L , Chen LL . RNA circles with minimized immunogenicity as potent PKR inhibitors. Mol Cell. 2022;82(2):420‐434.e6. 10.1016/j.molcel.2021.11.019 34951963

[29] Hsiue EH , Wright KM , Douglass J , et al. Targeting a neoantigen derived from a common TP53 mutation. Science. 2021;371(6533):eabc8697. 33649166 10.1126/science.abc8697 PMC8208645

[30] Poole A , Karuppiah V , Hartt A , et al. Therapeutic high affinity T cell receptor targeting a KRAS(G12D) cancer neoantigen. Nat Commun. 2022;13(1):5333. 36088370 10.1038/s41467-022-32811-1 PMC9464187

[31] Amaya L , Grigoryan L , Li Z , et al. Circular RNA vaccine induces potent T cell responses. Proc Natl Acad Sci U S A. 2023;120(20):e2302191120. 37155869 10.1073/pnas.2302191120 PMC10193964

[32] Workman P , Aboagye EO , Balkwill F , et al. Guidelines for the welfare and use of animals in cancer research. Br J Cancer. 2010;102(11):1555‐1577. 20502460 10.1038/sj.bjc.6605642 PMC2883160

