# Supplementary material for: Circular RNA‐based neoantigen vaccine for hepatocellular carcinoma immunotherapy
Source: MedComm (2020). 2024 Jul 29;5(8):e667. doi: 10.1002/mco2.667 (PMC11286538; doi:10.1002/mco2.667)
Supplement: Supplementary file 1 — Supporting Information [file MCO2-5-e667-s001.docx]

**Circular RNA-based neoantigen vaccine for hepatocellular carcinoma immunotherapy**

Fei Wang^1,2,^*, Guang Cai^1,2,^*, Yingchao Wang^1,2^, Qiuyu Zhuang^1,2^, Zhixiong Cai^1,2^, Yingying Li^1,2^, Shaodong Gao^3^, Fang Li^3^, Cuilin Zhang^1,2^, Bixing Zhao^1,2,#^, Xiaolong Liu^1,2,#^

1 The United Innovation of Mengchao Hepatobiliary Technology Key Laboratory of Fujian Province, Mengchao Hepatobiliary Hospital of Fujian Medical University, Fuzhou 350025, P. R. China; Fujian Provincial Clinical Research Center for Hepatobiliary and Pancreatic Tumors, Fuzhou 350025, P. R. China

2 Mengchao Med-X Center, Fuzhou University, Fuzhou 350116, P. R. China

3 School of Basic Medical Sciences, Fujian Medical University, Fuzhou 350122, P. R. China

* These authors contributed equally

^#^ Correspondence:

Bixing Zhao ([bixingzhao@gmail.com](mailto:bixingzhao@gmail.com));

Xiaolong Liu ([xiaoloong.liu@gmail.com](mailto:xiaoloong.liu@gmail.com))

**Supplementary information**

**Supplementary Figure**

**
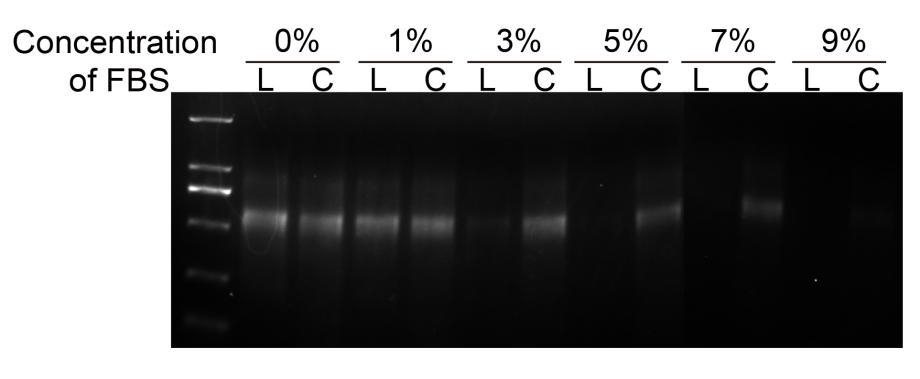
**

**Figure S1:** CircRNA (C) was more resistant to degradation by increasing concentrations of fetal bovine serum (FBS) than its linear RNA (L) at 37℃×30 min.

**
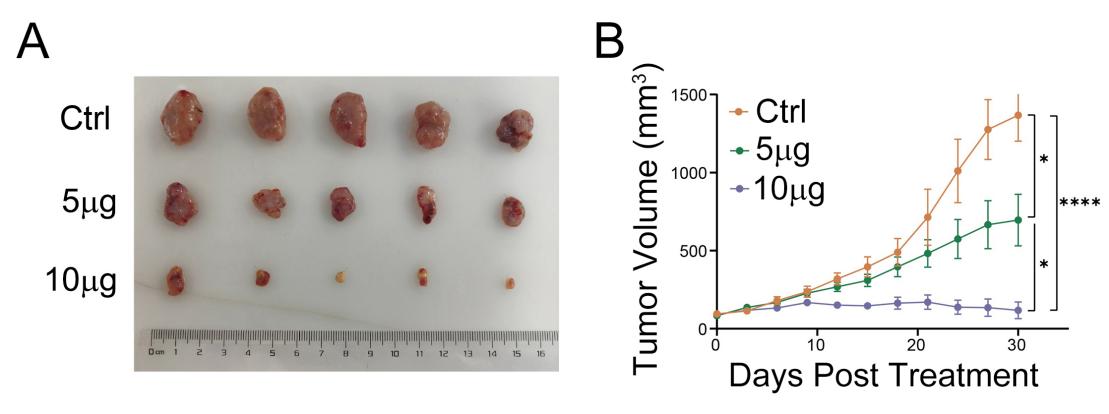
**

**Figure S2:** (A) Images of tumor size of mice after injection of Ctrl (CircRNA structures that do not express protein), 5 μg circRNA3×PTPN2, and 10 μg circRNA3×PTPN2 in an animal model of subcutaneous tumor in mice. (B) Statistics of tumor volume.

**
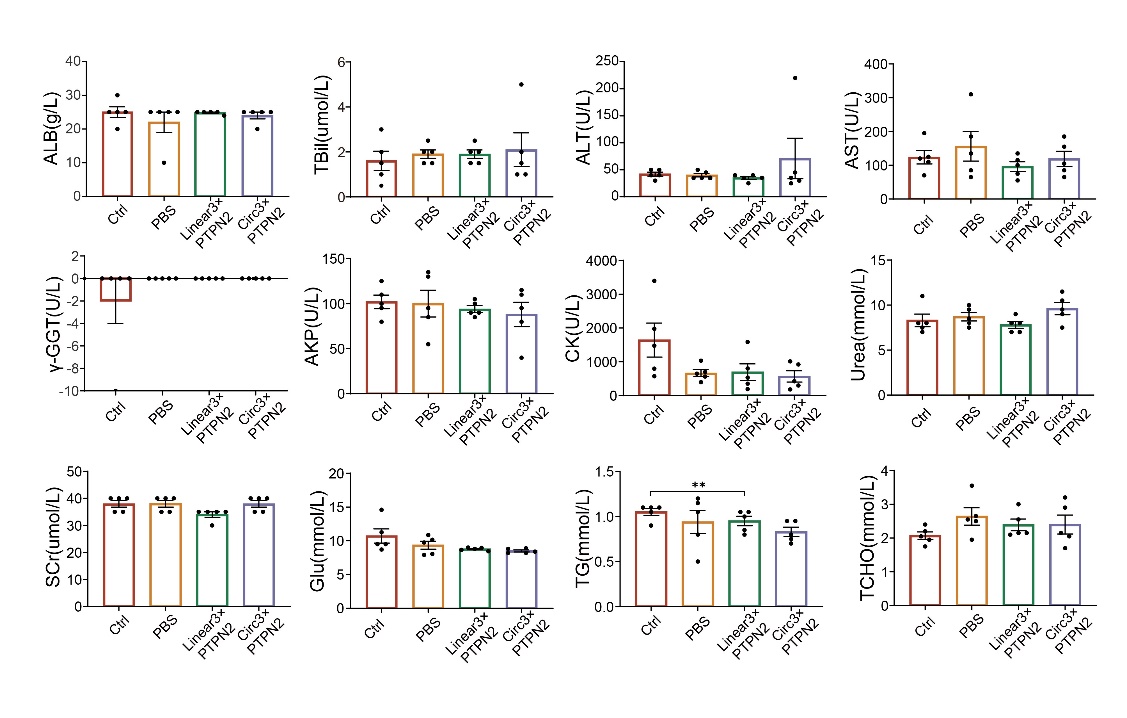
**

**Figure S3:** Mice blood plasma analysis for AKP (Alkaline phosphatase), ALT (Alanine aminotransferase), ALB (Albumin), CK (Creatine kinase), GLU (Glucose), AST (Aspartate aminotransferase), γ-GGT (Gamma-glutamyltransferase), SCr (Serum creatinine), TBil (Total bilirubin), TCHO (Total, cholesterol), Urea, TG (Triglyceride) after treat with PBS, linearRNA3×PTPN2, circRNA3×PTPN2.

**
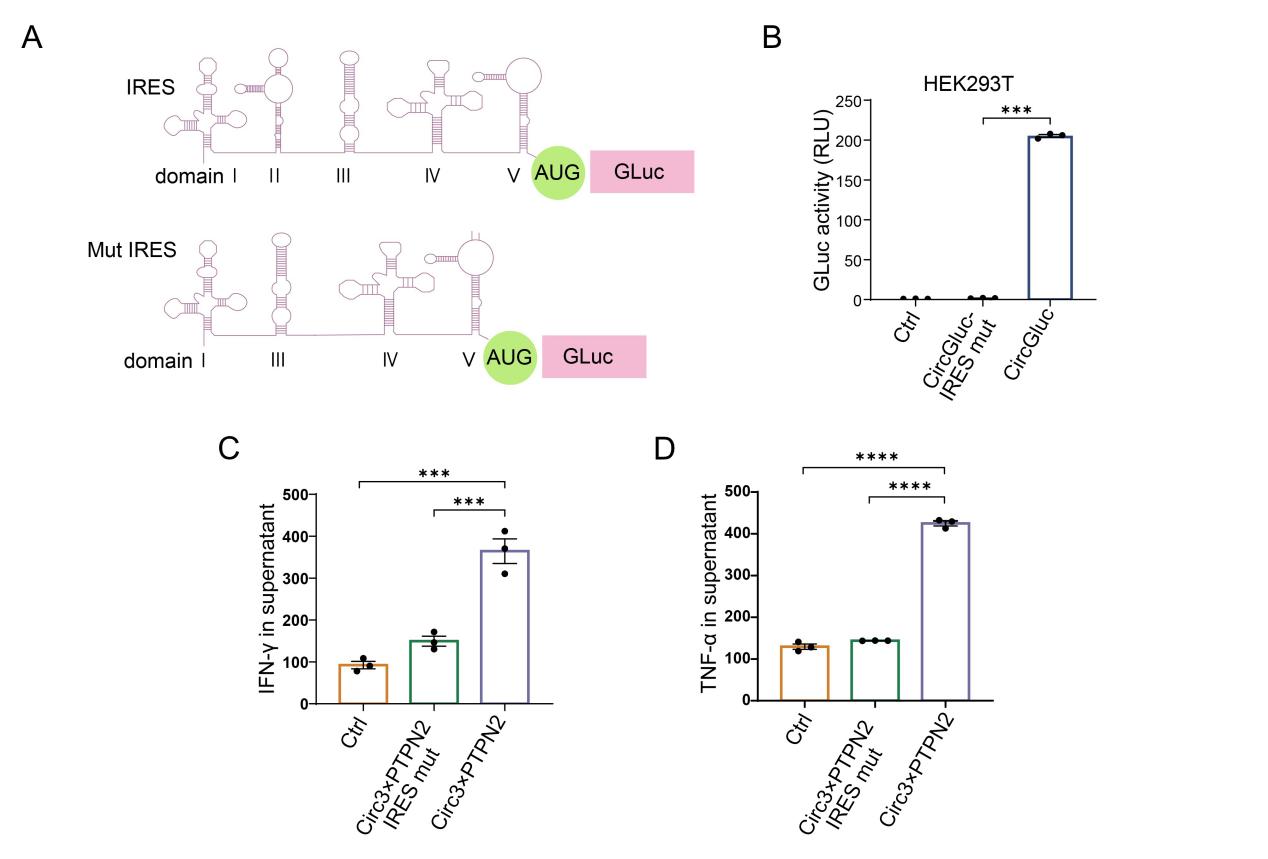
**

**Figure S4:** (A) Schematic diagram of the CVB3 IRES mutation. (B) Gaussia luciferase activity was detected after HEK293T transfection with different vector. (C-D) Detection of IFN-γ (C) and TNF-α (D) secretion in culture medium by Elisa method.

**Supplementary Table S1: Plasmid sequence**

| RT-linear-F | **CCATCCGGTGACTAATAGAGC** |
| --- | --- |
| RT-linear-R | **CCTCGCCAGACACGCTGAAC** |
| RT-circ-F | **CGGCATGGACGAGCTGTACAAG** |
| RT-circ-R | **GTGGGATCAACCCACAGGCTG** |
| CVB3 | **TTAAAACAGCCTGTGGGTTGATCCCACCCACAGGCCCATTGGGCGCTAGCACTCTGGTATCACGGTACCTTTGTGCGCCTGTTTTATACCCCCTCCCCCAACTGTAACTTAGAAGTAACACACACCGATCAACAGTCAGCGTGGCACACCAGCCACGTTTTGATCAAGCACTTCTGTTACCCCGGACTGAGTATCAATAGACTGCTCACGCGGTTGAAGGAGAAAGCGTTCGTTATCCGGCCAACTACTTCGAAAAACCTAGTAACACCGTGGAAGTTGCAGAGTGTTTCGCTCAGCACTACCCCAGTGTAGATCAGGTCGATGAGTCACCGCATTCCCCACGGGCGACCGTGGCGGTGGCTGCGTTGGCGGCCTGCCCATGGGGAAACCCATGGGACGCTCTAATACAGACATGGTGCGAAGAGTCTATTGAGCTAGTTGGTAGTCCTCCGGCCCCTGAATGCGGCTAATCCTAACTGCGGAGCACACACCCTCAAGCCAGAGGGCAGTGTGTCGTAACGGGCAACTCTGCAGCGGAACCGACTACTTTGGGTGTCCGTGTTTCATTTTATTCCTATACTGGCTGCTTATGGTGACAATTGAGAGATCGTTACCATATAGCTATTGGATTGGCCATCCGGTGACTAATAGAGCTATTATATATCCCTTTGTTGGGTTTATACCACTTAGCTTGAAAGAGGTTAAAACATTACAATTCATTGTTAAGTTGAATACAGCAAA** |
| PTPN2 | **AAAAGGTGGTTATATTGGCAACCTACTCTCACTAAGATGGGGTTTGTGTCA** |
| Gluc | **ATGGGAGTCAAAGTTCTGTTTGCCCTGATCTGCATCGCTGTGGCCGAGGCCAAGCCCACCGAGAACAACGAAGACTTCAACATCGTGGCCGTGGCCAGCAACTTCGCGACCACGGATCTCGATGCTGACCGCGGGAAGTTGCCCGGCAAGAAGCTGCCGCTGGAGGTGCTCAAAGAGATGGAAGCCAATGCCCGGAAAGCTGGCTGCACCAGGGGCTGTCTGATCTGCCTGTCCCACATCAAGTGCACGCCCAAGATGAAGAAGTTCATCCCAGGACGCTGCCACACCTACGAAGGCGACAAAGAGTCCGCACAGGGCGGCATAGGCGAGGCGATCGTCGACATTCCTGAGATTCCTGGGTTCAAGGACTTGGAGCCCATGGAGCAGTTCATCGCACAGGTCGATCTGTGTGTGGACTGCACAACTGGCTGCCTCAAAGGGCTTGCCAACGTGCAGTGTTCTGACCTGCTCAAGAAGTGGCTGCCGCAACGCTGTGCGACCTTTGCCAGCAAGATCCAGGGCCAGGTGGACAAGATCAAGGGGGCCGGTGGTGACTAA** |
| CVB3-EGFP | **CATATGGGGAGACCCTCGACCGTCGATTGTCCACTGGTCAACAATAGATGACTTACAACTAATCGGAAGGTGCAGAGACTCGACGGGAGCTACCCTAACGTCAAGACGAGGGTAAAGAGAGAGTCCAATTCTCAAAGCCAATAGGCAGTAGCGAAAGCTGCAAGAGAATGAAAATCCGTTGACCTTAAACGGTCGTGTGGGTTCAAGTCCCTCCACCCCCACGCCGGAAACGCAATAGCCGAAAAACAAAAAACAAAAAAAACAAAAAAAAAACCAAAAAAACAAAACACATTAAAACAGCCTGTGGGTTGATCCCACCCACAGGCCCATTGGGCGCTAGCACTCTGGTATCACGGTACCTTTGTGCGCCTGTTTTATACCCCCTCCCCCAACTGTAACTTAGAAGTAACACACACCGATCAACAGTCAGCGTGGCACACCAGCCACGTTTTGATCAAGCACTTCTGTTACCCCGGACTGAGTATCAATAGACTGCTCACGCGGTTGAAGGAGAAAGCGTTCGTTATCCGGCCAACTACTTCGAAAAACCTAGTAACACCGTGGAAGTTGCAGAGTGTTTCGCTCAGCACTACCCCAGTGTAGATCAGGTCGATGAGTCACCGCATTCCCCACGGGCGACCGTGGCGGTGGCTGCGTTGGCGGCCTGCCCATGGGGAAACCCATGGGACGCTCTAATACAGACATGGTGCGAAGAGTCTATTGAGCTAGTTGGTAGTCCTCCGGCCCCTGAATGCGGCTAATCCTAACTGCGGAGCACACACCCTCAAGCCAGAGGGCAGTGTGTCGTAACGGGCAACTCTGCAGCGGAACCGACTACTTTGGGTGTCCGTGTTTCATTTTATTCCTATACTGGCTGCTTATGGTGACAATTGAGAGATCGTTACCATATAGCTATTGGATTGGCCATCCGGTGACTAATAGAGCTATTATATATCCCTTTGTTGGGTTTATACCACTTAGCTTGAAAGAGGTTAAAACATTACAATTCATTGTTAAGTTGAATACAGCAAAATGGTGAGCAAGGGCGAGGAGCTGTTCACCGGGGTGGTGCCCATCCTGGTCGAGCTGGACGGCGACGTAAACGGCCACAAGTTCAGCGTGTCTGGCGAGGGCGAGGGCGATGCCACCTACGGCAAGCTGACCCTGAAGTTCATCTGCACCACCGGCAAGCTGCCCGTGCCCTGGCCCACCCTCGTGACCACCCTGACCTACGGCGTGCAGTGCTTCAGCCGCTACCCCGACCACATGAAGCAGCACGACTTCTTCAAGTCCGCCATGCCCGAAGGCTACGTCCAGGAGCGCACCATCTTCTTCAAGGACGACGGCAACTACAAGACCCGCGCCGAGGTGAAGTTCGAGGGCGACACCCTGGTGAACCGCATCGAGCTGAAGGGCATCGACTTCAAGGAGGACGGCAACATCCTGGGGCACAAGCTGGAGTACAACTACAACAGCCACAACGTCTATATCATGGCCGACAAGCAGAAGAACGGCATCAAGGCGAACTTCAAGATCCGCCACAACATCGAGGACGGCAGCGTGCAGCTCGCCGACCACTACCAGCAGAACACCCCCATCGGCGACGGCCCCGTGCTGCTGCCCGACAACCACTACCTGAGCACCCAGTCCGCCCTGAGCAAAGACCCCAACGAGAAGCGCGATCACATGGTCCTGCTGGAGTTCGTGACCGCCGCCGGGATCACTCTCGGCATGGACGAGCTGTACAAGTAAAAAAAACAAAAAACAAAACGGCTATTATGCGTTACCGGCGAGACGCTACGGACTTAAATAATTGAGCCTTAAAGAAGAAATTCTTTAAGTGGATGCTCTCAAACTCAGGGAAACCTAAATCTAGTTATAGACAAGGCAATCCTGAGCCAAGCCGAAGTAGTAATTAGTAAGACCAGTGGACAATCGACGGATAACAGCATATCTAGGGATCC** |
| CVB3-Gluc | **TAATACGACTCACTATAGGGGGAGACCCTCGACCGTCGATTGTCCACTGGTCAACAATAGATGACTTACAACTAATCGGAAGGTGCAGAGACTCGACGGGAGCTACCCTAACGTCAAGACGAGGGTAAAGAGAGAGTCCAATTCTCAAAGCCAATAGGCAGTAGCGAAAGCTGCAAGAGAATGAAAATCCGTTGACCTTAAACGGTCGTGTGGGTTCAAGTCCCTCCACCCCCACGCCGGAAACGCAATAGCCGAAAAACAAAAAACAAAAAAAACAAAAAAAAAACCAAAAAAACAAAACACATTAAAACAGCCTGTGGGTTGATCCCACCCACAGGCCCATTGGGCGCTAGCACTCTGGTATCACGGTACCTTTGTGCGCCTGTTTTATACCCCCTCCCCCAACTGTAACTTAGAAGTAACACACACCGATCAACAGTCAGCGTGGCACACCAGCCACGTTTTGATCAAGCACTTCTGTTACCCCGGACTGAGTATCAATAGACTGCTCACGCGGTTGAAGGAGAAAGCGTTCGTTATCCGGCCAACTACTTCGAAAAACCTAGTAACACCGTGGAAGTTGCAGAGTGTTTCGCTCAGCACTACCCCAGTGTAGATCAGGTCGATGAGTCACCGCATTCCCCACGGGCGACCGTGGCGGTGGCTGCGTTGGCGGCCTGCCCATGGGGAAACCCATGGGACGCTCTAATACAGACATGGTGCGAAGAGTCTATTGAGCTAGTTGGTAGTCCTCCGGCCCCTGAATGCGGCTAATCCTAACTGCGGAGCACACACCCTCAAGCCAGAGGGCAGTGTGTCGTAACGGGCAACTCTGCAGCGGAACCGACTACTTTGGGTGTCCGTGTTTCATTTTATTCCTATACTGGCTGCTTATGGTGACAATTGAGAGATCGTTACCATATAGCTATTGGATTGGCCATCCGGTGACTAATAGAGCTATTATATATCCCTTTGTTGGGTTTATACCACTTAGCTTGAAAGAGGTTAAAACATTACAATTCATTGTTAAGTTGAATACAGCAAAATGGGAGTCAAAGTTCTGTTTGCCCTGATCTGCATCGCTGTGGCCGAGGCCAAGCCCACCGAGAACAACGAAGACTTCAACATCGTGGCCGTGGCCAGCAACTTCGCGACCACGGATCTCGATGCTGACCGCGGGAAGTTGCCCGGCAAGAAGCTGCCGCTGGAGGTGCTCAAAGAGATGGAAGCCAATGCCCGGAAAGCTGGCTGCACCAGGGGCTGTCTGATCTGCCTGTCCCACATCAAGTGCACGCCCAAGATGAAGAAGTTCATCCCAGGACGCTGCCACACCTACGAAGGCGACAAAGAGTCCGCACAGGGCGGCATAGGCGAGGCGATCGTCGACATTCCTGAGATTCCTGGGTTCAAGGACTTGGAGCCCATGGAGCAGTTCATCGCACAGGTCGATCTGTGTGTGGACTGCACAACTGGCTGCCTCAAAGGGCTTGCCAACGTGCAGTGTTCTGACCTGCTCAAGAAGTGGCTGCCGCAACGCTGTGCGACCTTTGCCAGCAAGATCCAGGGCCAGGTGGACAAGATCAAGGGGGCCGGTGGTGACTAAAAAAAACAAAAAACAAAACGGCTATTATGCGTTACCGGCGAGACGCTACGGACTTAAATAATTGAGCCTTAAAGAAGAAATTCTTTAAGTGGATGCTCTCAAACTCAGGGAAACCTAAATCTAGTTATAGACAAGGCAATCCTGAGCCAAGCCGAAGTAGTAATTAGTAAGACCAGTGGACAATCGACGGATAACAGCATATCTAG** |
| CVB3-PTPN2-Gluc | **GGGAGACCCTCGACCGTCGATTGTCCACTGGTCAACAATAGATGACTTACAACTAATCGGAAGGTGCAGAGACTCGACGGGAGCTACCCTAACGTCAAGACGAGGGTAAAGAGAGAGTCCAATTCTCAAAGCCAATAGGCAGTAGCGAAAGCTGCAAGAGAATGAAAATCCGTTGACCTTAAACGGTCGTGTGGGTTCAAGTCCCTCCACCCCCACGCCGGAAACGCAATAGCCGAAAAACAAAAAACAAAAAAAACAAAAAAAAAACCAAAAAAACAAAACACATTAAAACAGCCTGTGGGTTGATCCCACCCACAGGCCCATTGGGCGCTAGCACTCTGGTATCACGGTACCTTTGTGCGCCTGTTTTATACCCCCTCCCCCAACTGTAACTTAGAAGTAACACACACCGATCAACAGTCAGCGTGGCACACCAGCCACGTTTTGATCAAGCACTTCTGTTACCCCGGACTGAGTATCAATAGACTGCTCACGCGGTTGAAGGAGAAAGCGTTCGTTATCCGGCCAACTACTTCGAAAAACCTAGTAACACCGTGGAAGTTGCAGAGTGTTTCGCTCAGCACTACCCCAGTGTAGATCAGGTCGATGAGTCACCGCATTCCCCACGGGCGACCGTGGCGGTGGCTGCGTTGGCGGCCTGCCCATGGGGAAACCCATGGGACGCTCTAATACAGACATGGTGCGAAGAGTCTATTGAGCTAGTTGGTAGTCCTCCGGCCCCTGAATGCGGCTAATCCTAACTGCGGAGCACACACCCTCAAGCCAGAGGGCAGTGTGTCGTAACGGGCAACTCTGCAGCGGAACCGACTACTTTGGGTGTCCGTGTTTCATTTTATTCCTATACTGGCTGCTTATGGTGACAATTGAGAGATCGTTACCATATAGCTATTGGATTGGCCATCCGGTGACTAATAGAGCTATTATATATCCCTTTGTTGGGTTTATACCACTTAGCTTGAAAGAGGTTAAAACATTACAATTCATTGTTAAGTTGAATACAGCAAAATGAACTCCTTCTCCACAAGCGCCTTCGGTCCAGTTGCCTTCTCCCTGGGCCTGCTCCTGGTGTTGCCTGCTGCCTTCCCTGCCCCAAAAAGGTGGTTATATTGGCAACCTACTCTCACTAAGATGGGGTTTGTGTCAAGAGGCCGGAAGCGGAGGTCTATGGGAGTCAAAGTTCTGTTTGCCCTGATCTGCATCGCTGTGGCCGAGGCCAAGCCCACCGAGAACAACGAAGACTTCAACATCGTGGCCGTGGCCAGCAACTTCGCGACCACGGATCTCGATGCTGACCGCGGGAAGTTGCCCGGCAAGAAGCTGCCGCTGGAGGTGCTCAAAGAGATGGAAGCCAATGCCCGGAAAGCTGGCTGCACCAGGGGCTGTCTGATCTGCCTGTCCCACATCAAGTGCACGCCCAAGATGAAGAAGTTCATCCCAGGACGCTGCCACACCTACGAAGGCGACAAAGAGTCCGCACAGGGCGGCATAGGCGAGGCGATCGTCGACATTCCTGAGATTCCTGGGTTCAAGGACTTGGAGCCCATGGAGCAGTTCATCGCACAGGTCGATCTGTGTGTGGACTGCACAACTGGCTGCCTCAAAGGGCTTGCCAACGTGCAGTGTTCTGACCTGCTCAAGAAGTGGCTGCCGCAACGCTGTGCGACCTTTGCCAGCAAGATCCAGGGCCAGGTGGACAAGATCAAGGGGGCCGGTGGTGACTAAAAAAAACAAAAAACAAAACGGCTATTATGCGTTACCGGCGAGACGCTACGGACTTAAATAATTGAGCCTTAAAGAAGAAATTCTTTAAGTGGATGCTCTCAAACTCAGGGAAACCTAAATCTAGTTATAGACAAGGCAATCCTGAGCCAAGCCGAAGTAGTAATTAGTAAGACCAGTGGACAATCGACGGATAACAGCATATCTAG** |
| CVB3-3xPTPN2 | **GGGAGACCCTCGACCGTCGATTGTCCACTGGTCAACAATAGATGACTTACAACTAATCGGAAGGTGCAGAGACTCGACGGGAGCTACCCTAACGTCAAGACGAGGGTAAAGAGAGAGTCCAATTCTCAAAGCCAATAGGCAGTAGCGAAAGCTGCAAGAGAATGAAAATCCGTTGACCTTAAACGGTCGTGTGGGTTCAAGTCCCTCCACCCCCACGCCGGAAACGCAATAGCCGAAAAACAAAAAACAAAAAAAACAAAAAAAAAACCAAAAAAACAAAACACATTAAAACAGCCTGTGGGTTGATCCCACCCACAGGCCCATTGGGCGCTAGCACTCTGGTATCACGGTACCTTTGTGCGCCTGTTTTATACCCCCTCCCCCAACTGTAACTTAGAAGTAACACACACCGATCAACAGTCAGCGTGGCACACCAGCCACGTTTTGATCAAGCACTTCTGTTACCCCGGACTGAGTATCAATAGACTGCTCACGCGGTTGAAGGAGAAAGCGTTCGTTATCCGGCCAACTACTTCGAAAAACCTAGTAACACCGTGGAAGTTGCAGAGTGTTTCGCTCAGCACTACCCCAGTGTAGATCAGGTCGATGAGTCACCGCATTCCCCACGGGCGACCGTGGCGGTGGCTGCGTTGGCGGCCTGCCCATGGGGAAACCCATGGGACGCTCTAATACAGACATGGTGCGAAGAGTCTATTGAGCTAGTTGGTAGTCCTCCGGCCCCTGAATGCGGCTAATCCTAACTGCGGAGCACACACCCTCAAGCCAGAGGGCAGTGTGTCGTAACGGGCAACTCTGCAGCGGAACCGACTACTTTGGGTGTCCGTGTTTCATTTTATTCCTATACTGGCTGCTTATGGTGACAATTGAGAGATCGTTACCATATAGCTATTGGATTGGCCATCCGGTGACTAATAGAGCTATTATATATCCCTTTGTTGGGTTTATACCACTTAGCTTGAAAGAGGTTAAAACATTACAATTCATTGTTAAGTTGAATACAGCAAAATGAACTCCTTCTCCACAAGCGCCTTCGGTCCAGTTGCCTTCTCCCTGGGCCTGCTCCTGGTGTTGCCTGCTGCCTTCCCTGCCCCAAAAAGGTGGTTATATTGGCAACCTACTCTCACTAAGATGGGGTTTGTGTCAAGAGGCCGGAAGCGGAGGTCTAAAAGGTGGTTATATTGGCAACCTACTCTCACTAAGATGGGGTTTGTGTCAAGAGGCCGGAAGCGGAGGTCTAAAAGGTGGTTATATTGGCAACCTACTCTCACTAAGATGGGGTTTGTGTCAAAAAAACAAAAAACAAAACGGCTATTATGCGTTACCGGCGAGACGCTACGGACTTAAATAATTGAGCCTTAAAGAAGAAATTCTTTAAGTGGATGCTCTCAAACTCAGGGAAACCTAAATCTAGTTATAGACAAGGCAATCCTGAGCCAAGCCGAAGTAGTAATTAGTAAGACCAGTGGACAATCGACGGATAACAGCATATCTAG** |
